# Transcriptomic Analysis of the Brown Planthopper, *Nilaparvata lugens*, at Different Stages after *Metarhizium anisopliae* Challenge

**DOI:** 10.3390/insects11020139

**Published:** 2020-02-24

**Authors:** Yifan Peng, Jifeng Tang, Jiaqin Xie

**Affiliations:** 1 Genetic Engineering Research Center, School of Life Sciences, Chongqing University, Chongqing 401331, China; 2 Chongqing Engineering Research Center for Fungal Insecticides/Key Laboratory of Gene Function and Regulation Technology under Chongqing Municipal Education Commission, Chongqing 401331, China

**Keywords:** *Metarhizium anisopliae*, transcriptomic analysis, *Nilaparvata lugens*, initial infection, pest control

## Abstract

*Nilaparvata lugens* is one of the major pests of rice and results in substantial yield loss every year. Our previous study found that the entomopathogenic fungus *Metarhizium anisopliae* showed effective potential for controlling this pest. However, the mechanisms underlying *M. anisopliae* infection of *N. lugens* are not well known. In the present study, we further examined the transcriptome of *N. lugens* at 4 h, 8 h, 16 h, and 24 h after *M. anisopliae* infection by Illumina deep sequencing. In total, 174.17 Gb of data was collected after sequencing, from which 23,398 unigenes were annotated by various databases, including 3694 newly annotated genes. The results showed that there were 246 vs 75, 275 vs 586, 378 vs 1055, and 638 vs 182 up- and downregulated differentially expressed genes (DEGs) at 4 h, 8 h, 16 h, and 24 h after *M. anisopliae* infection, respectively. The biological functions and associated metabolic processes of these genes were determined with the Clusters of Orthologous Groups (COG), Gene Ontology (GO), and Kyoto Encyclopedia of Genes and Genomes (KEGG) databases. The DEGs data were verified using RT-qPCR. These results indicated that the DEGs during the initial fungal infection appropriately reflected the time course of the response to the fungal infection. Taken together, the results of this study provide new insights into the molecular mechanisms underlying the insect host response to fungal infection, especially during the initial stage of infection, and may improve the potential control strategies for *N. lugens*.

## 1. Introduction

The brown planthopper *Nilaparvata lugens* (Hemiptera: Delphacidae) is one of the most dominant pests of rice worldwide [1,2]. This pest has severe negative effects on rice production, and outbreak areas reached 1.2 million hectares in 2010 in South China alone [3]. *N. lugens* feeds by sucking rice phloem sap and may result in rice death directly [4]. This planthopper also transmits viruses, such as the rice ragged stunt virus and rice grassy stunt virus, which further cause rice “grassy stunt” and “ragged stunt” diseases and impact rice growth, even causing mortality [5,6]. Many attempts have been made to suppress the occurrence of *N. lugens* in rice paddies. Among the methods used, chemical insecticides, including imidacloprid, thiamethoxam, and pymetrozine, are the major controls used [1,7,8]. However, side effects on nontarget natural enemies and the impact of insecticides on the environment have received much attention and sparked increasing interest in decreasing insecticide use [5,9]. On the other hand, transgenic methods and resistant rice varieties have also been used to defend against rice pests, but such methods may not provide desirable results in actual paddy fields [10,11,12].

Compared to chemical insecticides, microbial control shows many benefits in terms of efficiency and less environmental risks [13,14]. The entomopathogenic fungi *Beauveria bassiana* and *M. anisopliae* are the two most commonly used biological control agents against insect pests and have achieved good results [15,16]. For instance, *B. bassiana* can be used to control insect pests, such as *Helicoverpa armigera* [17], *Atta cephalotes* [18], and *Alphitobius diaperinus* [19], and *M. anisopliae* can be used to control insect pests such as *Locusta migratoria* [20], *Chironomus riparius* [21], *Zeugodacus cucurbitae* [22], and *Meccus pallidipennis* [23]. Moreover, these entomopathogenic fungi also show good potential for the control of insecticide-resistant pests, which may be due to their specific infection mechanisms [24,25,26]. Several reports have shown the development of transgenic fungi for insect control with greater efficiency than that of naturally occurring fungi [27]. The virulence of fungi can be enhanced substantially by the integration of genes such as the insecticidal scorpion toxin (Bjα IT) [28,29]. Although these entomopathogenic fungal agents have been used to control different insect pest species, there are few desirable fungal strains for rice planthopper control. However, *M. anisopliae* CQMa421 is one such strain that was recently described by our research group [30].

Conidial adhesion and detoxification of entomopathogenic fungi on insect host cuticles are vital processes in the initiation of infection. Certain proteins (MAD1, G-protein-coupled receptors (GPCRs), dehydrogenases, and lipases) and pathways (mitogen-activated protein kinase (MAPK) and protein kinase A (PKA) pathways) are involved in such processes [31,32]. After host penetration, rapid proliferation of fungal hyphal bodies in the insect host haemocoel may deprive the host of nutrients and result in the death of the insect host [31]. In contrast, the insect host may sense external substances when encountering pathogens or viruses. After the initial infection by pathogens, the host may activate serial physiological responses to defend against such infection [33,34]. Although invertebrates lack an adaptive immune response, they may defend against pathogens by relying on their innate immunity (i.e., cellular and humoral immune responses) [35,36,37]. In hosts, these xenobiotics can be countered by phagocytosis or by the activation of host innate immunity. A few studies have reported the physiological responses of insect hosts to pathogen infection by analyzing host enzymatic activities and gene expression levels or by transcriptomic profiling [33,38,39,40]. Such transcriptomic analysis is a widely used method to study the effects of environmental stressors, including UV exposure [41], insecticidal stress [40,42], and entomopathogenic stress [39], on different insect species. These studies have provided useful insights into the underlying mechanisms and interactions of hosts and pathogens or substances. However, few reports have explored the interactions between the fungus *M. anisopliae* and *N. lugens*, although *M. anisopliae* shows potential for infecting planthoppers.

In our previous study, we found that the fungal strain CQMa421 may infect adults and nymphs of *N. lugens*, indicating a potential control strategy for this pest [30]. Although a few studies have examined the transcriptome of *N. lugens* in response to insecticides and at different developmental stages [43,44], such analyses during fungal infection have scarcely been performed. Transcriptomic analyses have provided the foundation for understanding stress resistance and indicated that a few genes, such as genes encoding P450s and acetylcholinesterase, may be involved in these responses [42,44]. To better understand the mechanisms underlying *M. anisopliae* infection of *N. lugens*, we further studied the *N. lugens* responses to *M. anisopliae* infection for different periods by transcriptomic analysis. This study provides new insights for further study of insect host responses to fungal infection, especially in the initial stage, and may improve the potential control strategies for *N. lugens*.

## 2. Methods and Materials

### 2.1. Insect Culture and Fungal Treatment

The rice planthopper *N. lugens* was originally obtained from Nanjing University (Nanjing, China) in 2017 and was maintained in the Plant Experimental Base at Chongqing University, Chongqing, China. The individuals of *N. lugens* were reared on fresh rice seedlings at 27 ± 1 °C with a light:dark (L:D) photoperiod of 14:10 h. In this study, the nymphs of *N. lugens* were randomly collected and treated with *M. anisopliae*. Prior to the experiments, 10 rice seedlings were placed in each column bucket (10 mm × 150 mm diameter × height) and were treated using a 1 × 10^8^ conidia/mL suspension of *M. anisopliae* (prepared by the methods described in our previous study) [30]. After this treatment for 2 h, the *N. lugens* nymphs were transferred onto the rice seedlings and incubated in a bioassay room at 27 ± 1 °C and 14:10 h (L:D). Then, 60 nymphs from both the treated and control groups were collected at 4 h, 8 h, 16 h, and 24 h after incubation for analysis of the *M. anisopliae* infection-induced transcriptome. Control groups were subjected to the same handling procedures without fungal exposure. The eight sample groups were flash frozen in liquid nitrogen and stored prior to RNA extraction. All treatments were carried out in triplicate.

### 2.2. Total RNA Isolation, Quantification, and Sequencing

Total RNA was extracted from whole *N. lugens* nymphs for the eight groups using TRIzol reagent (Invitrogen, Carlsbad, CA, USA). The purity, concentration, and integrity of RNA samples were tested using a NanoPhotometer^®^ spectrophotometer (IMPLEN, Westlake Village, CA, USA) and agarose gels to ensure the use of high-quality samples for transcriptome sequencing. Then, a total amount of 1 μg of RNA per sample was used for RNA sample preparation. Sequencing libraries were generated using a NEBNext UltraTM RNA Library Prep Kit for Illumina (NEB, Ipswich, MA, USA) following the manufacturer’s recommendations, and index codes were added to attribute sequences to each sample. Briefly, mRNA was purified from total RNA using poly-T oligo-attached magnetic beads. Fragmentation was carried out using divalent cations under an elevated temperature in NEBNext First Strand Synthesis Reaction Buffer (5×). First-strand cDNA was synthesized using random hexamer primers and M-MuLV reverse transcriptase. Second-strand cDNA synthesis was subsequently obtained using DNA polymerase I and RNase H. The remaining overhangs were converted into blunt ends via exonuclease/polymerase activities. After adenylation of the 3’ ends of the DNA fragments, NEBNext adaptors with hairpin loop structures were ligated to prepare the samples for hybridization. To preferentially select cDNA fragments that were 240 bp in length, the library fragments were purified with the AMPure XP system (Beckman Coulter, Brea, CA, USA). Then, 3 μL of USER enzyme (NEB, Ipswich, MA, USA) was used with size-selected, adaptor-ligated cDNA at 37 °C for 15 min followed by 5 min at 95 °C before PCR. Then, PCR was performed with Phusion High-Fidelity DNA polymerase, universal PCR primers and the Index (X) primer. Finally, PCR products were purified by the AMPure XP system and library quality was assessed on an Agilent Bioanalyzer 2100 system (Agilent, Santa Clara, CA, USA).

Clustering of the index-coded samples was performed on a cBot Cluster Generation System using a TruSeq PE Cluster Kit v4-cBot-HS (Illumina, San Diego, CA, USA) according to the manufacturer’s instructions. After cluster generation, the library preparations were sequenced on an Illumina platform and paired-end reads were generated.

### 2.3. Data Analysis

Raw data/raw reads in FASTQ format were first processed through in-house Perl scripts. In this step, clean data/clean reads were obtained by removing reads containing adapters, reads containing poly-N sequences, and low-quality reads from the raw data. At the same time, the Q20, Q30, GC content and sequence duplication level of the clean data were calculated. All downstream analyses were based on clean data with high quality. The adaptor sequences and low-quality sequence reads were removed from the data sets. Raw sequences were transformed into clean reads after data processing. These clean reads were then mapped to the reference genome sequence [45]. Only reads with a perfect match or one mismatch were further analyzed and annotated based on the reference genome. Hisat2 software was used to map reads to the reference genome.

Gene functional annotation was based on the following databases: Nr (NCBI nonredundant protein sequences); Nt (NCBI nonredundant nucleotide sequences); Pfam (Protein family); KOG/COG (Clusters of Orthologous Groups of proteins); Swiss-Prot (a manually annotated and reviewed protein sequence database); KO (KEGG Orthologue database); and GO (Gene Ontology). Gene expression levels were estimated by the fragments per kilobase of transcript per million fragments mapped (FPKM) values with the following formula:

FPKM = cDNA Fragments/Mapped Fragments (Millions) ∗ Transcript Length (kb)

Differential expression analysis was performed using DESeq2, which provided statistical analyses for determining differential expression in digital gene expression (DGE) data using a model based on the negative binomial distribution. The resulting P values were adjusted using Benjamini and Hochberg’s approach for controlling the false discovery rate. Genes with an adjusted P value < 0.01 found by DESeq2 were considered differentially expressed. Gene Ontology (GO) enrichment analysis of the differentially expressed genes (DEGs) was implemented by the GOseq R package based on the Wallenius noncentral hypergeometric distribution, which adjusts for gene length bias in DEGs. KEGG is a database resource for understanding the high-level functions and utilities of biological systems, such as cells, organisms, and ecosystems, from molecular information, especially large-scale molecular datasets generated by genome sequencing and other high-throughput experimental technology (http://www.genome.jp/kegg/). KOBAS software was then used to test the statistical enrichment of DEGs in KEGG pathways.

### 2.4. Validation of DEG Libraries Using RT-qPCR

To validate the DEGs in the libraries, 20 DEGs (i.e., control vs treatment) were randomly selected for comparison using real-time quantitative PCR (RT-qPCR). RT-qPCR was performed on an iCycler iQ Real-time PCR System (Bio-Rad, Hercules, CA, USA) with a QuantiNove SYBR Green PCR Kit (QIAGEN, Dusseldorf, Germany) according to the manufacturer’s instructions. The cycling parameters were as follows: Initial denaturation at 95 °C for 10 s, followed by 40 cycles of 95 °C for 10 s, 56.5 °C for 20 s, and 72 °C for 20 s. The expression of 18S rRNA was selected for normalization of the expression of the DEGs according to the 2^−ΔΔ*C*t^ method. The primers designed for RT-qPCR in this experiment are listed in Table S1.

## 3. Results

### 3.1. Summary Evaluation of Digital Gene Expression

To obtain a global understanding of the transcriptomic response of *N. lugens* to fungal *M. anisopliae* infection for different periods, we constructed 24 DGE tag libraries. Such libraries showed the *N. lugens* response at 4 h, 8 h, 16 h, and 24 h after fungal infection, including the T-4 h vs W-4 h, T-8 h vs W-8 h, T-16 h vs W-16 h, and T-24 h vs W-24 h comparisons for the treatment group and control group for different periods. In total, 41.25–77.51 million raw reads were obtained from each treatment sample (Table 1). Prior to mapping, low-quality and adapter reads were filtered, and 20.63–38.75 million clean sequence reads per library were retained (Table 1). All samples had Q30 values greater than 89.66%, and the GC content ranged from 39.35% to 42.62% (Table 1). More than 52.59%–63.84% of the reads from all the libraries were uniquely mapped on the *N. lugens* genome, and a few reads showed multiple mapping (Figure S1, Table S2).

### 3.2. Transcriptomic Comparison and Analysis of Different Initial Infection Stages

**Figure 1:**
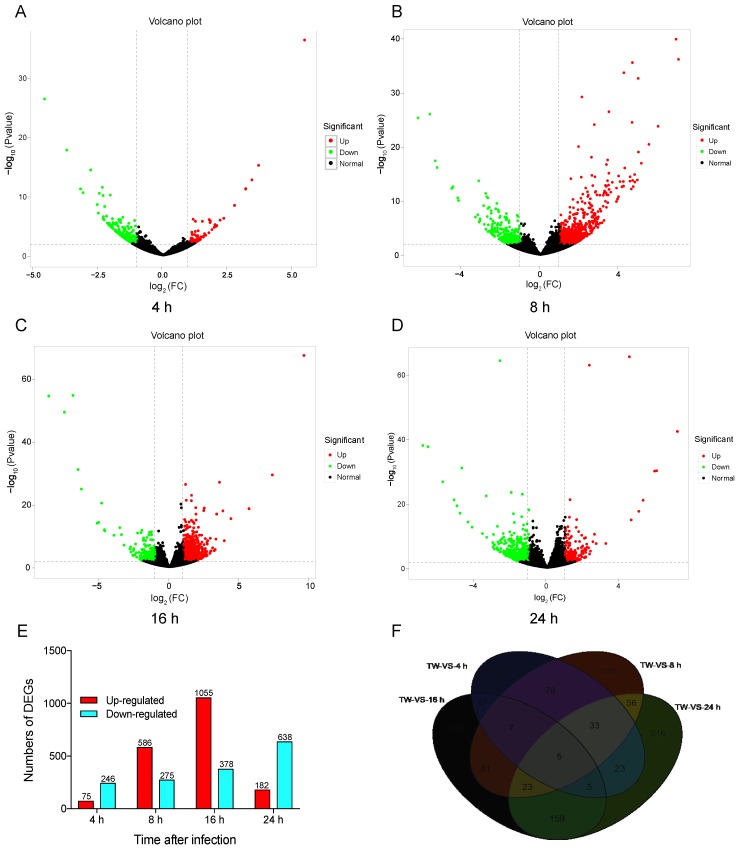
Volcano plot and number of differentially expressed genes (DEGs) after challenge with *M. anisopliae* at different periods. **A**: Volcano plot of DEGs at 4 h; **B**: Volcano plot of DEGs at 8 h; **C**: Volcano plot of DEGs at 16 h; **D**: Volcano plot of DEGs at 24 h; **E**: Numbers of DEGs at different period; **F**: Venn diagram of DEGs at 4 h, 8 h, 16 h, and 24 h post-infection.

To compare the DEGs among different libraries, the gene expression levels were first determined form the FPKM values. Global analysis of the transcriptomic changes in *N. lugens* at different time points after *M. anisopliae* infection demonstrated up- or downregulated genes in the control and treatment groups. DESeq.2 was selected to test DEGs with *p* < 0.01. These results showed that 75 genes were upregulated at 4 h after fungal infection, and 246 genes were downregulated by over two-fold (|log_2_ (FoldChange)| > 2) (Figure 1A). At 8 h, there were 586 upregulated and 275 downregulated genes (Figure 1B). At 16 h, there were 1055 upregulated and 378 downregulated genes (Figure 1C). There were 182 upregulated and 638 downregulated genes (Figure 1D) after infection for 24 h. These results showed that different genes may be involved in infection of *N. lugens* by *M. anisopliae* over time. We also found 149, 578, 1132, and 516 DEGs specifically expressed at 4 h, 8 h, 16 h, and 24 h, respectively, and 5 DEGs were commonly expressed during this period (Figure 1E,F).

### 3.3. Functional Classification and Pathway Analysis

**Figure 2:**
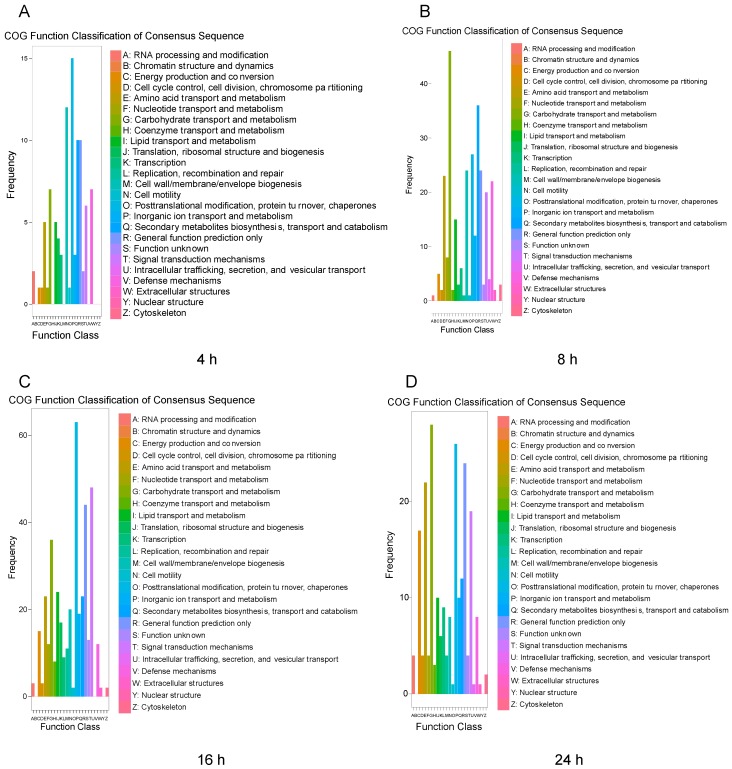
Clusters of orthologous groups of proteins (COG) classification of *N. lugens* DEGs after challenge with *M. anisopliae* at different periods. **A**: COG classification of *N. lugens* DEGs after challenge with *M. anisopliae* at 4 h; **B**: COG classification of *N. lugens* DEGs after challenge with *M. anisopliae* at 8 h; **C**: COG classification of *N. lugens* DEGs after challenge with *M. anisopliae* at 16 h; **D**: COG classification of *N. lugens* DEGs after challenge with *M. anisopliae* at 24 h.

**Figure 3:**
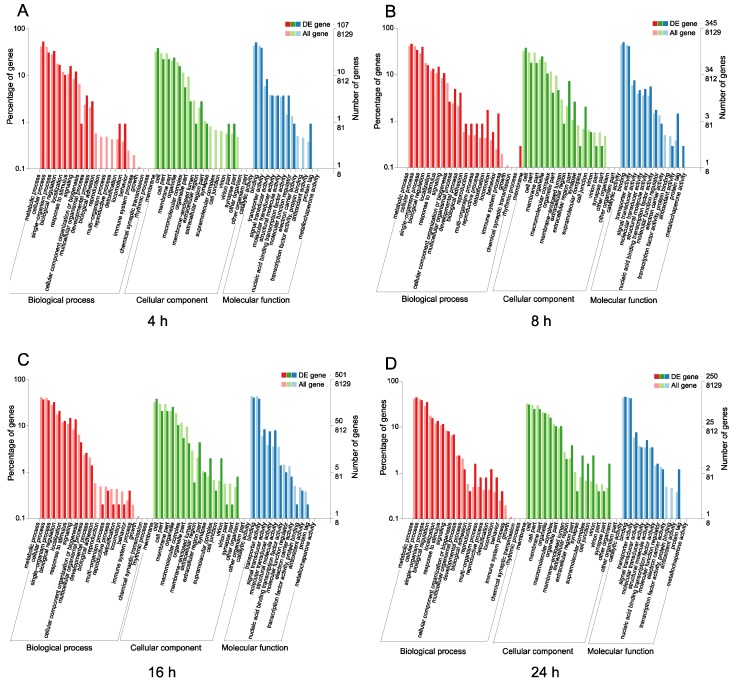
Gene Ontology (GO) categories of *N. lugens* DEGs after challenge with *M. anisopliae* at different periods. **A**: GO categories of *N. lugens* DEGs after challenge with *M. anisopliae* at 4 h; **B**: GO categories of *N. lugens* DEGs after challenge with *M. anisopliae* at 8 h; **C**: GO categories of *N. lugens* DEGs after challenge with *M. anisopliae* at 16 h; **D**: GO categories of *N. lugens* DEGs after challenge with *M. anisopliae* at 24 h.

To examine the functions of the DEGs after challenge with *M. anisopliae*, we used the COG and GO databases to map their different functions of the DEGs. In the COG database, a total of 2865 protein sequences were matched and divided into 26 categories. For different infection periods, the COG database showed different abundances. Specifically, at 4 h, the biological function category was assigned to 321 DEGs (Figure 2A). After infection for 8 h, the biological function category was assigned to 861 DEGs, and after 16 h (Figure 2B), the biological function category was assigned to 1433 DEGs (Figure 2C). However, the biological function category included 820 DEGs after 24 h, indicating different abundances over time (Figure 2D). The annotated GO terms included 2685 DEGs (321, 861, 1433, and 820 DEGs) in the BLAST database in the categories of biological process, cell component and molecular function (Figure 3). For different infection periods, each treatment also showed different abundances among the categories.

**Figure 4:**
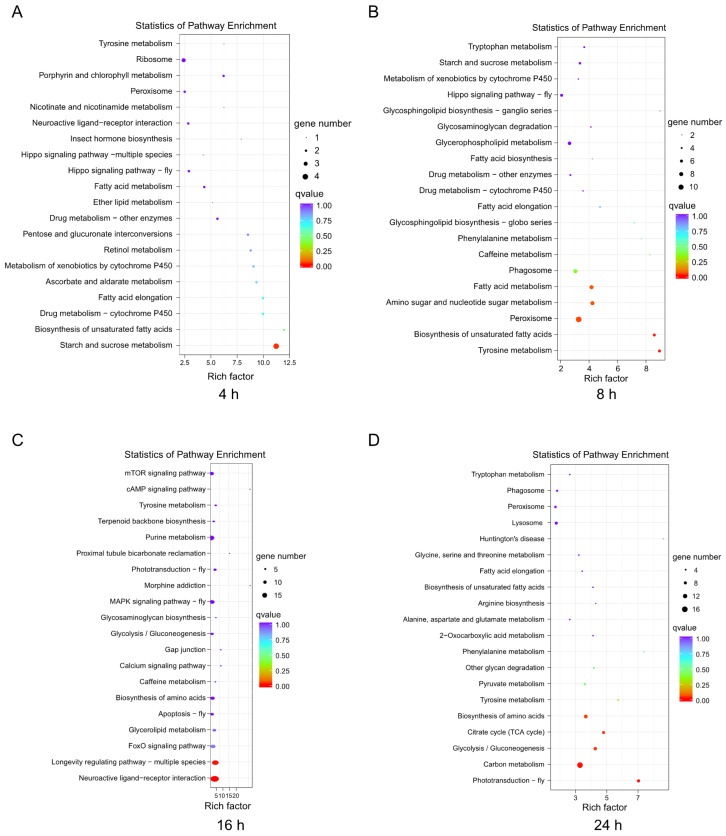
Enrichment and dispersion point map of differentially expressed genes (DEGs) in KEGG pathways. **A**: The DEGs involved in the pathways after challenge for 4 h; **B**: The DEGs involved in the pathways after challenge for 8 h; **C**: The DEGs involved in the pathways after challenge for 16 h; **D**: The DEGs involved in the pathways after challenge for 24 h. The circles in the graph indicate the KEGG pathway with the name and enrichment factor are displayed on the y- and x-axes, respectively.

In this study, we selected KEGG to identify the metabolic and signal transduction pathways associated with the DEGs. There were 57 DEGs, which mapped to 39 pathways in the KEGG database between the control and treatment groups at the 4-h infection time point (Figure 4A), and 221 DEGs, which mapped to 86 pathways in the KEGG database between the control and treatment groups at 8 h post-infection (Figure 4B). In contrast, 328 DEGs between the control and treatment groups at 16 h post-infection were mapped to 108 pathways in the KEGG database (Figure 4C), and 228 DEGs between the control and treatment groups at 24 h post-infection were mapped to 75 pathways in the KEGG database (Figure 4D). The top 20 pathways in the richness analysis are displayed in Figure 4, including the top pathways of starch and sucrose metabolism (4 proteins) at 4 h, peroxisome (11 proteins) at 8 h, neuroactive ligand-receptor interaction (18 proteins) and purine metabolism (15 proteins) at 16 h, and carbon metabolism (16 proteins) and endocytosis (6 proteins) at 24 h.

### 3.4. Validation of DEGs Using RT-qPCR

**Figure 5:**
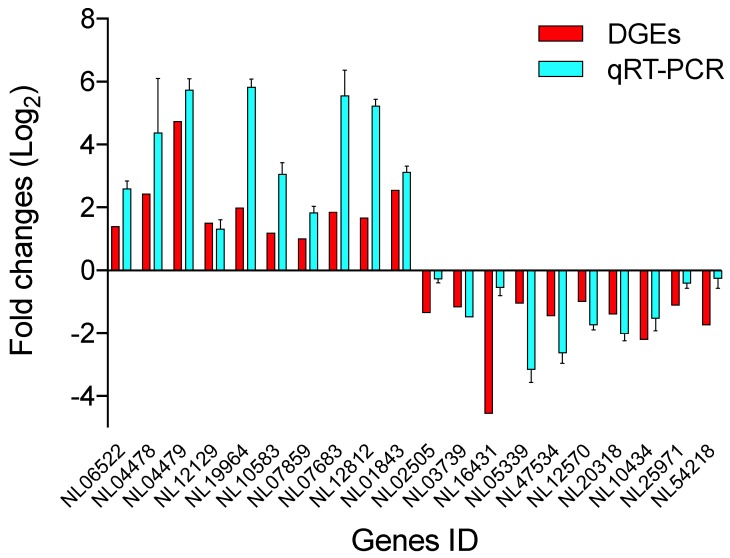
Transcriptomic validation by RT-qPCR and RNA-seq of the genes.

To verify the mRNA data obtained by RNA-seq, 20 randomly selected DEGs (10 upregulated and 10 downregulated) were analyzed by RT-qPCR. The results exhibited similar expression patterns as those of the DGE analysis (Figure 5), indicating that the DGE results in this study were reliable.

## 4. Discussion

The resistance of *N. lugens* to many chemical insecticides has received much attention and increased the interest in reducing insecticide use [46,47,48]. Microbial control shows effective potential for replacing/reducing the use of chemical insecticides for the control of insect pests, such as *Z. cucurbitae* [22], *L. migratoria* [49], and *H. armigera* [50]. The entomopathogenic fungus *M. anisopliae*, as an important biological control agent, can be used to control many insect pests, including the rice pest *N. lugens,* and suppress populations of this pest in field conditions [30]. Although the control efficiency of these entomopathogenic fungi against insect pests has been well evaluated, the underlying mechanisms of such infections in *N. lugens* are less known. Thus, we analyzed the digital gene expression profile of *N. lugens* by high-throughput sequencing after challenge with *M. anisopliae*, providing insights for further studies of the mechanisms underlying the interaction between insect hosts and entomopathogenic fungi.

These results identified DEGs under different periods after infection from 4 h to 24 h. Certain metabolic processes and pathways, including pathogen recognition, energy metabolism, immune responses, and defense, were involved in the responses to *M. anisopliae* infection. In general, the cuticle of the insect host is the first protective barrier against infection [31]. Moreover, the recognition of pathogens by hosts is an important stage for the defense against infection [31,51]. During this period, pattern recognition molecules, such as peptidoglycan recognition proteins (PGRPs), β-1,3-glucan recognition proteins (βGRPs), galectins, C-type lectins (CTLs), and scavenger receptors (SCRs) [39,52,53], play vital roles. These groups of proteins may recognize and respond to invading pathogens [39]. In our study, infection by *M. anisopliae* may have stimulated the upregulation of insect cuticle protein (Gene ID 06250, 07046 and 06902; Tables S3 and S4) expression after 4 h. Moreover, several common responses were observed after *M. anisopliae* infection, including the suppression of metabolic pathways (lipid and amino acid metabolism, Gene ID15475 and 05753; Tables S3 and S4) and an elevated expression of several genes related to insect hormone biosynthesis (Gene ID 10544; Table S3) and cholesterol ester synthesis (Gene ID 01794; Table S3). However, we also noticed that the signal involved in the immune responses (unregulated peroxidase, Gene ID 01845 and spaetzle, Gene ID 13168; Table S4) was activated at 4 h post-infection. Such processes included the lysosome, endocytosis, and oxidative phosphorylation pathways. In contrast, the responses of *L. migratoria* to fungal infection were activated at 8 h post-infection.

Different substances may cause distinct insect host recognition responses. From the eight groups, libraries were constructed to analyse the transcriptome at 4 h, 8 h, 16 h, and 24 h post-infection. In the initial infection process, the fungus adheres to the body of the insect host, and many enzymes hydrolyse the host cuticle [31]. A few pathways, including the Toll, IMD, JAK-STAT, and PPO pathways, may activate immune responses to defend against external pathogens [31,51,54]. The entomopathogenic fungus may invade the host body by penetrating the cuticle [31]. Infection of *N. lugens* with *M. anisopliae* caused the cuticle-, cell activity-, immunity-, and energy-related gene differential expression to be sustained for 24 h, but differences were observed over time. After 8 h of infection, genes related to nucleotide transport (Gene ID 15029; Table S3) and a few secondary metabolite biosynthesis pathways (Gene ID 16876 and 19778; Table S3) exhibited the highest differential expression, while the juvenile hormone in the haemolymph (Gene ID 07487, 07486 and 07483; Table S3) was significantly suppressed at this time. At 16 h and 24 h, phospholipase B (Gene ID 17474; Table S3) and defensin-related genes (Gene ID 04479; Table S3) had high expression, but the expression of trypsin (Gene ID 08061; Table S3) and alcohol dehydrogenase (Gene ID 15752; Table S3) was significantly inhibited.

The DEGs included metabolic functions involving starch and sucrose metabolism, peroxisome, neuroactive ligand-receptor interaction, purine metabolism, and carbon metabolism. However, the different infection processes of pathogens may be involved in specific pathways and show some functional differences [33,55]. Entomopathogenic fungi first settled in the cuticle of the insect host and secrete enzymes, including chitinase and proteolytic enzymes, which triggered a host response. At 4 h post-infection, 321 genes were differentially expressed, most of which were involved in starch/sucrose metabolism and xenobiotic metabolism. A previous study reported that the insect host *L. migratoria* showed immune responses at 4 h after fungal infection [33]. Proteins such as the βGRP and haemocytin play important roles in recognizing pathogens and other substances [39,53]. In our study, we found that at 4 h post-infection, the specific pathways of insect hormone biosynthesis and ribosome and neuroactive ligand-receptor interaction may have been involved in the response to fungal infection in the early stage. After 24 h post-infection, the up- and downregulated genes (182 and 638 up- and downregulated genes, respectively; Figure 1E) were more abundant than those at 16 h of infection (1055 and 378 up- and downregulated genes, respectively; Figure 1E). These difference in the results may be attributed to the different periods were associated with distinct host responses to pathogenic infection. Several other studies in insect species have also shown temporal expression patterns [54,55].

In conclusion, we evaluated the transcriptome of *N. lugens* following *M. anisopliae* infection for different periods by DEG analysis. We found and annotated total of 26,587 genes, including 6783 newly upregulated genes and 3694 newly downregulated genes. These results showed that fungal infection could result in the regulation of metabolic processes and immune responses after 4 h. This study provides insights into the underlying physiological responses of insect hosts to fungal infection and may improve control strategies for *N. lugens*.

## Figures and Tables

**Table 1:** Statistical summary for *M. anisopliae*-infected and control groups.

Samples	Clean Reads	Total Reads	GC Content	%≥Q30
T-4h-1	21,547,204	43,094,408	42.41%	90.35%
T-4h-2	22,512,422	45,024,844	39.92%	90.86%
T-4h-3	24,148,619	48,297,238	42.26%	90.63%
T-8h-1	38,754,441	77,508,882	42.62%	91.54%
T-8h-2	24,002,510	48,005,020	41.32%	90.02%
T-8h-3	22,122,639	44,245,278	39.74%	91.17%
T-16h-1	23,547,935	47,095,870	42.30%	91.52%
T-16h-2	22,069,447	44,138,894	40.20%	91.42%
T-16h-3	22,239,750	44,479,500	42.57%	90.65%
T-24h-1	25,170,248	50,340,496	42.54%	89.66%
T-24h-2	26,533,253	53,066,506	41.57%	90.98%
T-24h-3	22,340,639	44,681,278	41.71%	91.47%
W-4h-1	24,392,303	48,784,606	41.81%	90.56%
W-4h-2	25,368,822	50,737,644	41.82%	90.73%
W-4h-3	23,584,970	47,169,940	41.91%	90.75%
W-8h-1	21,703,936	43,407,872	44.07%	90.44%
W-8h-2	27,725,344	55,450,688	44.13%	89.82%
W-8h-3	31,397,538	62,795,076	44.10%	90.28%
W-16h-1	21,417,269	42,834,538	40.71%	89.96%
W-16h-2	24,218,605	48,437,210	39.80%	91.23%
W-16h-3	20,903,499	41,806,998	39.35%	90.55%
W-24h-1	22,905,768	45,811,536	42.11%	91.31%
W-24h-2	23,248,816	46,497,632	40.75%	90.19%
W-24h-3	20,628,223	41,256,446	41.82%	91.05%

T: *M. anisopliae* treatment group; W: Control group.

## Supplementary Materials

The following are available online at https://www.mdpi.com/2075-4450/11/2/139/s1, Figure S1: Matches of *N. lugens* transcriptome unigenes with respect to other species, Table S1: Primers used for RT-qPCR in this study, Table S2: Statistical summary of *N. lugens* after challenge with *M. anisopliae*, Table S3 and S4: the information of DEGs involving in this study.
