# Supplementary material for: Transcriptomic Analysis of the Brown Planthopper, Nilaparvata lugens, at Different Stages after Metarhizium anisopliae Challenge
Source: Insects. 2020 Feb 24;11(2):139. doi: 10.3390/insects11020139 (PMC7073985; doi:10.3390/insects11020139)
Supplement: Supplementary file 1 [file insects-11-00139-s001.pdf]

Supplementary Figure 1. Matches of *N. lugens* transcriptome unigenes with respect to other species.

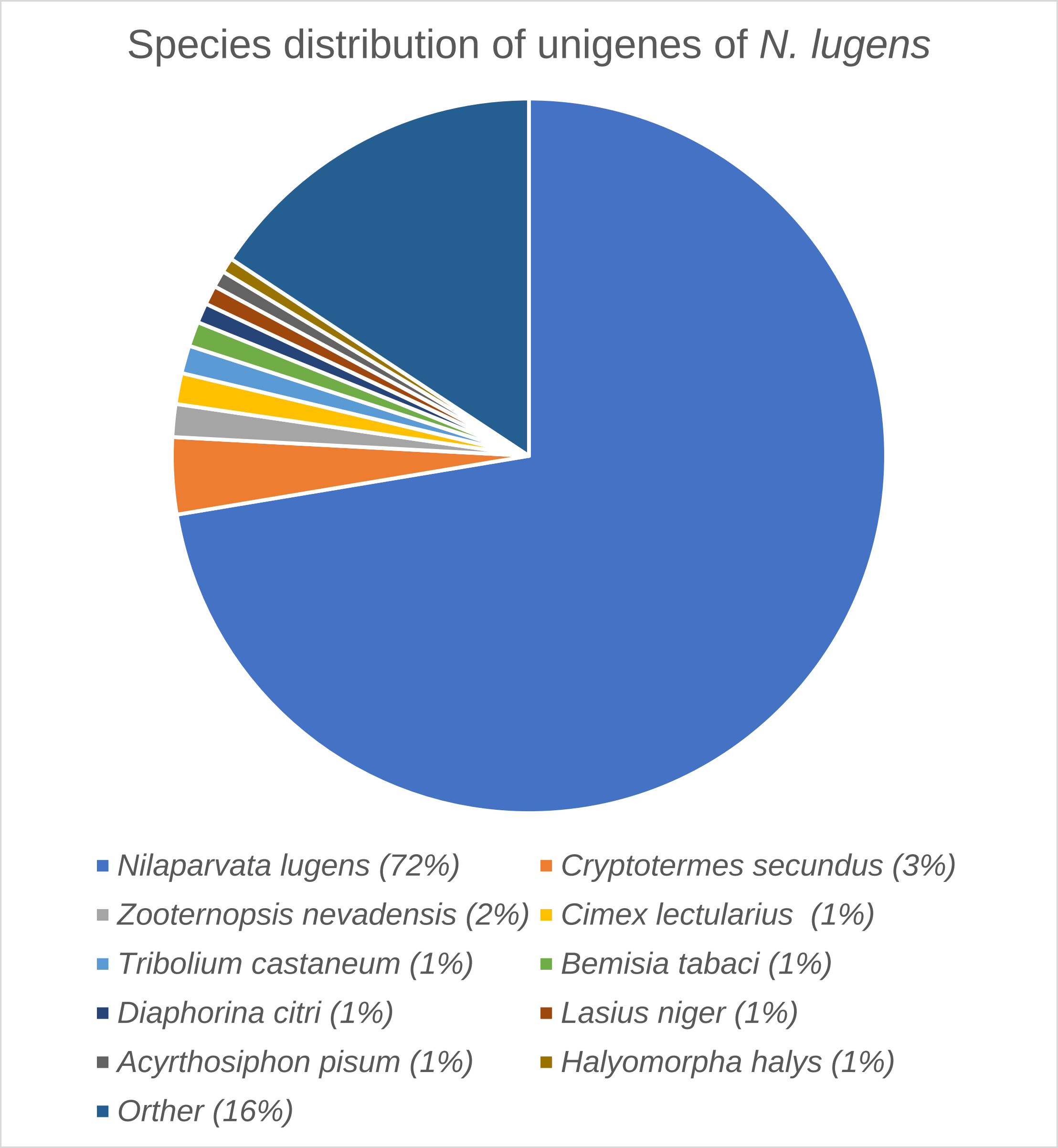

Supplementary Table 1. Primers used for RT-qPCR in this study.

Gene ID	Primer directions	Primer sequences (5'-3')
NL06522	Forward	ACCACAACAACATTCAGT
	Reverse	AGTCCACAGTCTACCAAT
NL04478	Forward	ATTCTTCCATTACTGCTGTT
	Reverse	AGTTGGTAGACTGTTCAC
NL04479	Forward	TAGTTGCCTCTGTAATGG
	Reverse	CCTTCTTGCCTCTGATTA
NL12129	Forward	AATCCACCATCAAGACATT
	Reverse	CCACTATCGCTTCATACC
NL19964	Forward	CGCTGGAAGTCTCATTAG
	Reverse	TTGTTATTGGTATCGCTGTA
NL10583	Forward	CAATGGAAGTGGAAGTGG
	Reverse	CACGCATCATCTGGTAAG
NL07859	Forward	AGTCGCTGTCTTCCTATTG
	Reverse	GTCATACCAACGCTCCTT
NL07683	Forward	ATGGAAGATGTTGATGATGAT
	Reverse	AAGAGAAGCACCTGAGAA
NL12812	Forward	CTTACGGTATCCCTCTCAATA
	Reverse	CTTCCTGGAGTATTACAATTCT
NL08143	Forward	TGGCAAGAATCCTCAAGA
	Reverse	ACAGAGACGGAGTTATCG
NL02505	Forward	GTGAATGATGATGTAGAATGG
	Reverse	TAAGAGGAGTGTGAGTGA
NL03739	Forward	AAGACCGTATCATCAAGT
	Reverse	TACTGTATTGTTCCTTACCT
NL16431	Forward	ACTCTATCCACCTCTACC
	Reverse	TCACTTCCATTCATCTTCA
NL05339	Forward	CGCTCTCAACAACAATGG

	Reverse	CAACCGCTACTAACAACAT
NL47534	Forward	TTCTGTCATCGGTCACTA
	Reverse	TTGCGGTCATTGTCATAA
NL12570	Forward	GTAGAGTTGAGGAGTAGGATA
	Reverse	GCTATACCGATGTTATGGAA
NL20318	Forward	CCGACATTAGCACTGATA
	Reverse	TACCAAACATTCCAAGATTT
NL10434	Forward	ACAGATTAGGTTCACTTCAA
	Reverse	CATTCTACACTGCGTTCT
NL25071	Forward	GAATGTCCGATTGTAGATGAA
	Reverse	TGGCTGATATTATGGTGAGA
NL54218	Forward	GGTCTTGTATGGTCTTCA
	Reverse	CAGTTTCTTCTTTAGTTTATCAG
18S rRNA	Forward	CGCTA CTACCGATTGAA
	Reverse	GGAAA CCTTGTTACGACTT

Supplementary Table 2. Output statistics of *N. lugens* after challenge with *M. anisopliae*

Samples	Mapped Reads	Uniq Mapped Reads	Multiple Map Reads	Reads Map to '+'	Reads Map to '-'
T4-1	32,468,737 (75.34%)	26,058,440 (60.47%)	6,410,297 (14.88%)	13,807,628 (32.04%)	13,873,397 (32.19%)
T4-2	33,800,347 (75.07%)	25,106,153 (55.76%)	8,694,194 (19.31%)	13,346,344 (29.64%)	13,433,760 (29.84%)
T4-3	37,109,410 (76.84%)	30,073,398 (62.27%)	7,036,012 (14.57%)	15,925,291 (32.97%)	15,999,162 (33.13%)
T8-1	58,583,658 (75.58%)	46,491,797 (59.98%)	12,091,861 (15.60%)	24,754,488 (31.94%)	24,819,683 (32.02%)
T8-2	35,099,897 (73.12%)	28,359,521 (59.08%)	6,740,376 (14.04%)	15,029,378 (31.31%)	15,223,391 (31.71%)
T8-3	34,292,855 (77.51%)	27,034,592 (61.10%)	7,258,263 (16.40%)	14,365,221 (32.47%)	14,411,949 (32.57%)
T16-1	36,327,142 (77.13%)	29,636,425 (62.93%)	6,690,717 (14.21%)	15,736,225 (33.41%)	15,929,448 (33.82%)
T16-2	34,110,482 (77.28%)	27,116,685 (61.43%)	6,993,797 (15.84%)	14,335,632 (32.48%)	14,475,811 (32.80%)
T16-3	33,215,067 (74.68%)	27,477,148 (61.77%)	5,737,919 (12.90%)	14,493,887 (32.59%)	14,610,131 (32.85%)
T24-1	37,265,496 (74.03%)	30,275,178 (60.14%)	6,990,318 (13.89%)	16,128,627 (32.04%)	16,194,401 (32.17%)
T24-2	40,065,113 (75.50%)	32,279,891 (60.83%)	7,785,222 (14.67%)	16,995,605 (32.03%)	17,091,684 (32.21%)
T24-3	33,873,017 (75.81%)	27,050,146 (60.54%)	6,822,871 (15.27%)	14,335,642 (32.08%)	14,392,362 (32.21%)
W4-1	38,239,390 (78.38%)	31,253,974 (64.07%)	6,985,416 (14.32%)	16,628,246 (34.09%)	16,820,729 (34.48%)

W4-2	39,586,193 (78.02%)	32,391,994 (63.84%)	7,194,199 (14.18%)	17,213,210 (33.93%)	17,353,211 (34.20%)
W4-3	34,595,028 (73.34%)	27,988,722 (59.34%)	6,606,306 (14.01%)	14,873,238 (31.53%)	14,934,512 (31.66%)
W8-1	31,830,944 (73.33%)	25,964,100 (59.81%)	5,866,844 (13.52%)	13,665,560 (31.48%)	13,796,047 (31.78%)
W8-2	42,192,811 (76.09%)	34,787,972 (62.74%)	7,404,839 (13.35%)	18,405,199 (33.19%)	18,512,792 (33.39%)
W8-3	47,641,097 (75.87%)	38,429,680 (61.20%)	9,211,417 (14.67%)	20,277,649 (32.29%)	20,509,346 (32.66%)
W16-1	32,438,023 (75.73%)	25,916,225 (60.50%)	6,521,798 (15.23%)	13,636,627 (31.84%)	13,760,969 (32.13%)
W16-2	37,443,588 (77.30%)	29,146,875 (60.17%)	8,296,713 (17.13%)	15,539,646 (32.08%)	15,629,584 (32.27%)
W16-3	28,926,110 (69.19%)	21,986,361 (52.59%)	6,939,749 (16.60%)	11,693,772 (27.97%)	11,716,569 (28.03%)
W24-1	35,445,871 (77.37%)	28,607,912 (62.45%)	6,837,959 (14.93%)	15,282,539 (33.36%)	15,323,744 (33.45%)
W24-2	35,032,367 (75.34%)	27,004,925 (58.08%)	8,027,442 (17.26%)	14,363,216 (30.89%)	14,479,585 (31.14%)
W24-3	31,708,845 (76.86%)	25,780,849 (62.49%)	5,927,996 (14.37%)	13,657,266 (33.10%)	13,770,704 (33.38%)

T, *M. anisopliae* treatment group; W, control group.

Table 3. Top twenty up- and down-regulated DEGs in *N. lugens* after *M. anisopliae* challenge.

Treatment	Gene ID	*P*-value	Fold Change (log2)	Up/Down regulated	Description
4 h after infection	NL40807	5.66E-37	5.46	up	Vitellogenin
	NL21487	7.77E-16	3.68	up	N/A
	NL58425	2.15E-13	3.42	up	ribosomal protein L3e, partial
	NL35745	4.40E-09	2.75	up	N/A
	NL49799	6.58E-07	2.34	up	N/A
	NL08709	1.33E-06	2.20	up	Protein RNA-directed DNA methylation 3
	NL06618	1.08E-05	2.07	up	N/A
	NL19138	1.46E-05	2.03	up	N/A
	NL19544	7.70E-06	1.99	up	60S ribosomal protein L23
	NL06292	2.18E-05	1.96	up	BTB/POZ domain-containing protein 6
	NL15475	5.24E-05	1.88	up	Vitellogenin
	NL16349	1.85E-06	1.82	up	Homeobox protein extradenticle
	NL56313	3.38E-06	1.81	up	Helicase
	NL49607	1.10E-06	1.77	up	Putative protein TPRXL
	NL03102	1.76E-04	1.76	up	Protein Wnt-16
	NL01832	4.70E-04	1.64	up	Vitellogenin
	NL20837	4.63E-04	1.62	up	Crustapain
	NL18677	2.52E-04	1.59	up	N/A
	NL05842	9.35E-04	1.55	up	N/A
	NL41498	7.33E-04	1.54	up	Sporozoite surface protein 2
	NL39423	4.88E-27	-4.59	down	N/A
	NL11553	2.08E-18	-3.73	down	Nose resistant to fluoxetine protein 6
	NL49423	3.11E-11	-3.10	down	N/A
	NL09618	4.62E-15	-2.80	down	WW domain-containing transcription regulator protein 1
	NL17550	3.12E-09	-2.55	down	Cuticle protein 16.5

	NL02377	8.72E-08	-2.51	down	N/A
	NL14111	4.11E-11	-2.47	down	FTZ-F1, partial
	NL14580	3.88E-12	-2.35	down	N/A
	NL06250	6.98E-07	-2.33	down	N/A
	NL20969	3.40E-07	-2.31	down	N/A
	NL20264	1.19E-06	-2.28	down	Odorant binding protein 3, partial
	NL01794	6.63E-09	-2.27	down	Nose resistant to fluoxetine protein 6
	NL10544	2.86E-07	-2.20	down	Cytochrome P450 18a1
	NL01946	4.27E-06	-2.16	down	Organic cation transporter protein
	NL18057	6.21E-06	-2.13	down	Ejaculatory bulb-specific protein 3
	NL11774	8.57E-06	-2.09	down	N/A
	NL33463	4.37E-06	-2.09	down	N/A
	NL07046	7.37E-06	-2.08	down	Cuticle protein 7
	NL06902	4.96E-06	-2.08	down	N/A
	NL13997	1.01E-05	-2.07	down	N/A
8 h after infection	NL07487	9.51E-37	7.04	up	Protein takeout
	NL07486	1.74E-40	6.91	up	Protein takeout
	NL07483	2.13E-24	5.99	up	Protein takeout
	NL31812	4.65E-21	5.53	up	Hypothetical protein RP20
	NL01946	1.47E-17	5.14	up	Organic cation transporter protein
	NL10607	1.22E-19	4.99	up	Cytochrome P450 4C1
	NL06225	3.08E-33	4.98	up	N/A
	NL05288	1.78E-14	4.94	up	N/A
	NL08289	4.21E-14	4.81	up	Protein takeout
	NL02377	1.76E-15	4.80	up	N/A
	NL07046	2.91E-13	4.73	up	Cuticle protein 7
	NL07554	3.70E-36	4.69	up	Pancreatic lipase-related protein 2
	NL07254	3.99E-25	4.67	up	Cysteine sulfinic acid decarboxylase
	NL16580	8.14E-13	4.63	up	N/A
	NL03036	4.48E-15	4.57	up	Cytochrome P450 4c3

	NL10849	2.94E-14	4.57	up	Nose resistant to fluoxetine protein 6
	NL11552	1.10E-16	4.30	up	Nose resistant to fluoxetine protein 6-like isoform X1
	NL09541	2.73E-34	4.25	up	UMP-CMP kinase
	NL07264	2.34E-13	4.24	up	La-related protein 6
	NL08760	3.36E-14	4.23	up	Cuticle protein 6
	NL15029	6.15E-26	-6.24	down	Adenylate kinase 7
	NL20821	1.18E-26	-5.64	down	N/A
	NL02642	8.80E-17	-5.27	down	N/A
	NL01356	6.10E-13	-4.52	down	Aftiphilin
	NL19169	1.09E-10	-4.18	down	N/A
	NL19699	1.26E-07	-3.32	down	N/A
	NL14009	4.76E-08	-3.20	down	N/A
	NL16876	2.51E-14	-3.15	down	Cytochrome P450 4g15
	NL11634	1.06E-06	-3.13	down	N/A
	NL20837	6.38E-07	-3.10	down	Crustapain
	NL19778	9.49E-10	-2.90	down	Cytochrome P450 4g15-like, partial
	NL07467	2.65E-06	-2.87	down	N/A
	NL05753	2.01E-05	-2.79	down	Trypsin-1
	NL15475	2.39E-05	-2.78	down	Vitellogenin
	NL00708	2.18E-11	-2.74	down	Esterase E4
	NL20598	9.96E-08	-2.71	down	N/A
	NL14010	3.35E-09	-2.68	down	Neuropeptide-like protein 31
	NL10970	3.61E-11	-2.68	down	Fatty acid synthase
	NL50364	5.96E-05	-2.67	down	N/A
	NL02647	5.83E-05	-2.66	down	Serine protease easter
16 h after infection	NL16850	5.48E-30	7.31	up	Serine/threonine-protein kinase kinX
	NL43859	3.10E-19	5.65	up	N/A
	NL20645	4.79E-16	4.36	up	Mitochondrial ornithine transporter 1-like, partial
	NL20116	5.14E-09	3.89	up	N/A

NL04506	1.70E-18	3.79	up	E3 ubiquitin-protein ligase RNF139
NL07467	1.71E-17	3.35	up	N/A
NL18745	3.12E-06	3.25	up	N/A
NL10891	7.98E-06	3.18	up	DnaJ homolog subfamily B member 13
NL08061	9.78E-07	3.06	up	Chymotrypsin-like protease CTRL
NL09626	1.25E-05	2.91	up	ATP-dependent RNA helicase DBP2-like, partial
NL14570	8.79E-10	2.89	up	Apolipoprotein D-like
NL20503	4.77E-09	2.71	up	Delta-like protein 1
NL11123	6.02E-08	2.68	up	Putative serine protease K12H4.7
NL10997	2.65E-04	2.67	up	Seminal fluid protein
NL03011	1.42E-08	2.65	up	Gamma-aminobutyric acid type B receptor subunit 2
NL14287	1.51E-05	2.65	up	Troponin C, isotype gamma
NL14645	3.89E-04	2.60	up	N/A
NL01957	1.10E-09	2.54	up	N/A
NL01389	1.62E-06	2.54	up	N/A
NL15414	2.25E-04	2.53	up	Putative serine protease K12H4.7
NL10213	2.96E-55	-6.88	down	N/A
NL20037	1.09E-31	-6.52	down	Inactive pancreatic lipase-related protein 1
NL20521	1.79E-25	-6.28	down	Exported protein (PHISTc), partial
NL17474	1.38E-14	-5.15	down	Putative phospholipase B-like 2
NL15044	5.59E-21	-4.84	down	Ubiquitin-40S ribosomal protein S27a
NL05659	3.86E-12	-4.62	down	N/A
NL11385	9.34E-11	-3.96	down	N/A
NL04821	3.50E-13	-3.55	down	Zinc finger protein 555
NL17823	6.16E-11	-3.43	down	Inactive pancreatic lipase-related protein 1
NL18162	1.30E-07	-3.24	down	Protein sine oculis-like, partial

	NL15422	2.19E-06	-2.82	down	Cyclin-dependent kinase 17
	NL17237	2.07E-05	-2.75	down	Muscle M-line assembly protein unc-89 isoform X5
	NL55478	2.69E-04	-2.61	down	Vacuole membrane protein 1-like, partial
	NL07385	7.29E-06	-2.58	down	N/A
	NL26851	5.75E-06	-2.57	down	N/A
	NL43998	1.66E-06	-2.55	down	Retrovirus-related Pol polyprotein from transposon 17.6
	NL45097	1.68E-04	-2.46	down	N/A
	NL15191	5.84E-08	-2.43	down	N/A
	NL20243	3.47E-05	-2.38	down	Kinase suppressor of Ras 2-like, partial
	NL07814	9.26E-04	-2.35	down	N/A
24 h after infection	NL54282	7.29E-43	7.22	up	Leukocyte receptor cluster member 8 homolog
	NL03938	1.26E-30	5.95	up	Kelch-like protein 5
	NL09021	4.01E-18	5.08	up	Protein winged eye
	NL16431	4.85E-66	4.56	up	Probable splicing factor, arginine/serine-rich 7
	NL03033	3.75E-08	3.25	up	N/A
	NL15752	4.03E-08	2.71	up	N/A
	NL19253	5.10E-11	2.62	up	UPF0598 protein
	NL48724	1.02E-08	2.53	up	Hypothetical protein BSL78_14642
	NL17447	8.65E-06	2.40	up	Inactive pancreatic lipase-related protein 1
	NL01160	7.08E-05	2.35	up	N/A
	NL07050	1.94E-63	2.34	up	N/A
	NL16631	8.50E-07	2.29	up	N/A
	NL10434	5.63E-07	2.21	up	BTB/POZ domain-containing protein 3
	NL16630	5.99E-08	2.19	up	L-2-aminoadipate reductase large subunit
	NL03439	3.44E-06	2.15	up	Zinc finger protein 77
	NL01501	4.97E-04	2.15	up	N/A

NL01841	6.48E-04	2.14	up	Lipoma HMGIC fusion partner
NL03625	4.92E-04	2.14	up	N/A
NL01921	2.09E-05	2.14	up	Sodium channel protein Nach
NL08247	4.99E-04	2.13	up	Lysosomal alpha-mannosidase
NL15662	3.84E-38	-6.62	down	Bromo adjacent homology domain-containing 1 protein
NL30575	1.04E-21	-5.17	down	Histone-lysine N-methyltransferase PRDM9
NL06659	7.47E-20	-5.01	down	Transcription factor SPT20 homolog, partial
NL32622	1.48E-17	-4.84	down	Protein CLP1 homolog isoform X1
NL04479	1.42E-31	-4.74	down	Defensin-like
NL16751	7.88E-15	-4.40	down	Hypothetical protein ALC60
NL19442	3.07E-11	-3.60	down	N/A
NL06681	6.30E-23	-3.38	down	Suppressor protein SRP40
NL03520	4.66E-10	-3.36	down	N/A
NL38390	5.89E-07	-3.00	down	N/A
NL14687	5.72E-11	-2.98	down	N/A
NL10088	1.48E-07	-2.96	down	Protein cueball
NL16364	1.94E-06	-2.93	down	Major facilitator superfamily domain-containing protein 6-A
NL08286	8.77E-08	-2.92	down	N/A
NL34455	2.18E-06	-2.78	down	N/A
NL10028	7.33E-07	-2.78	down	N/A
NL20829	4.50E-06	-2.73	down	N/A
NL09415	3.97E-06	-2.71	down	N/A
NL10248	4.56E-08	-2.69	down	N/A
NL09136	7.68E-65	-2.63	down	Roquin-1 isoform X1

N/A, No annotation.

Table 4. the information of other DEGs discussed in this study.

Gene ID	*P*-value	Fold Change (log2)	Up/Down regulated	Description
NL01845	8.43E-03	-1.20E+00	down	Peroxidase
NL07046	7.37E-06	2.08E+00	up	Insect cuticle protein
NL13168	1.65E-03	1.48E+00	up	Spaetzle
